# Supplementary material for: Learning through ferroelectric domain dynamics in solid-state synapses
Source: Nat Commun. 2017 Apr 3;8:14736. doi: 10.1038/ncomms14736 (PMC5382254; doi:10.1038/ncomms14736)
Supplement: Supplementary Information — Supplementary Figures [file ncomms14736-s1.pdf]

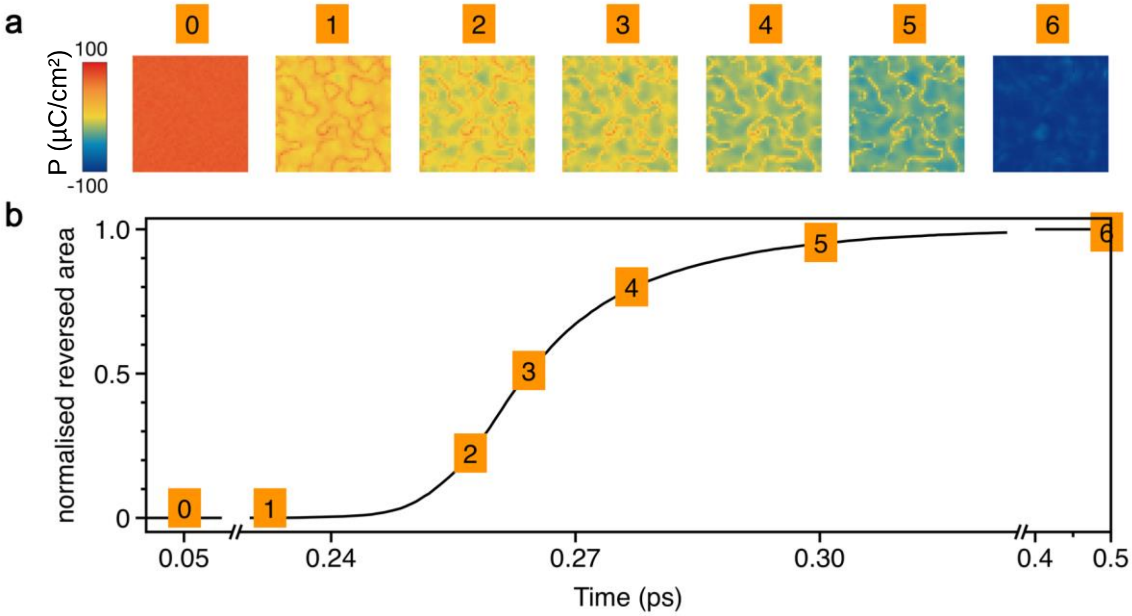

Supplementary Figure 1 | Polarisation switching predicted by effective Hamiltonian simulations.

(**a**) Evolution of the polarisation pattern along the [001] direction under a dc electric field of 2.5 V nm^{-1}.

(**b**) Normalised switched area as a function of time.

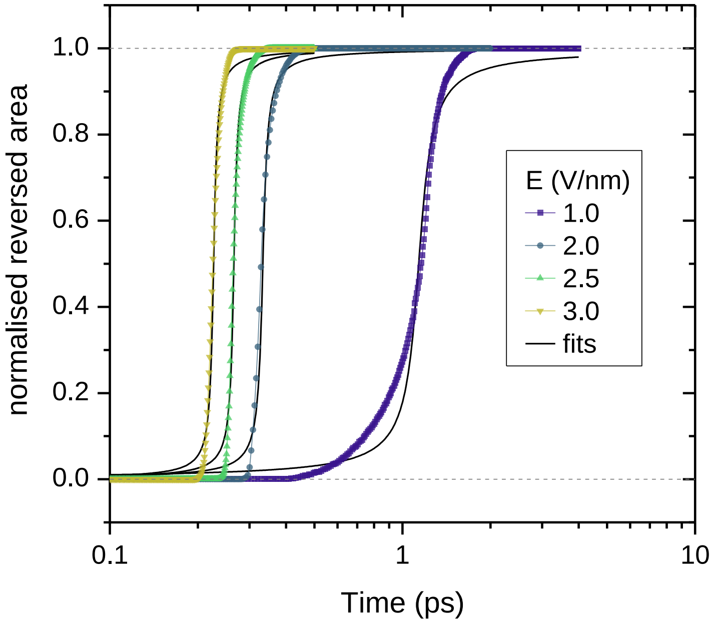

Supplementary Figure 2 | Switching dynamics as simulated by the effective Hamiltonian technique.

Normalised switched area as a function of time under various dc electric fields ranging from 1 to 3 V nm^{-1}. The black lines are fit results from the nucleation-limited switching model from Eq. 1.

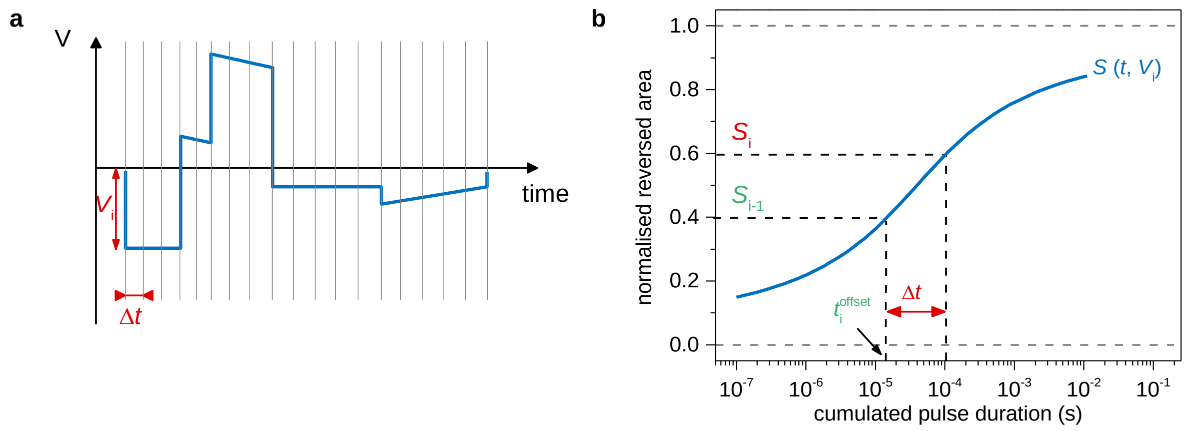

Supplementary Figure 3 | Modelling conductance variations under arbitrary voltage waveforms.

(**a**) Sketch of the voltage waveform divided in short segments of width Δt for which we assume a constant voltage (V_i). (**b**) Determination of the final state (S_i) resulting from the application of the pulse segment of voltage V_i, and length Δt. S_{i-1} corresponds to the polarisation state before the pulse. t_i^{offset} and S_i are computed from $S(V_i, t)$ curves from Eq. 1.
